# Effects of Fermented Bamboo Powder Supplementation on Serum Biochemical Parameters, Immune Indices, and Fecal Microbial Composition in Growing–Finishing Pigs

**DOI:** 10.3390/ani12223127

**Published:** 2022-11-13

**Authors:** Zhengqun Liu, Ning Li, Xiaoqiao Zhou, Zi Zheng, Chunhua Zhang, Shiyue Liang, Yuanming Li, Jun Yan, Qianjun Li, Shuqin Mu

**Affiliations:** 1 Institute of Animal Science and Veterinary, Tianjin Academy of Agriculture Sciences, Tianjin 300381, China; 2 Guangdong HAID Group Co., Ltd., Guangzhou 511442, China; 3 Liuyang Huanan Bamboo Industry Company, Liuyang 410300, China

**Keywords:** fermented bamboo powder, growth performance, serum indices, fecal microbiota, growing–finishing pigs

## Abstract

**Simple Summary:**

This study investigated the effects of fermented bamboo powder as a new feed ingredient by replacing wheat bran in swine diets. The results indicated that there were no negative effects on the growth performance, and that it exerted beneficial effects on promoting serum biochemical and immune indices, as well as modulating the fecal microbiota of pigs, when the wheat bran was replaced by fermented bamboo powder at 5% or 10% in the control diets. These results suggest that the fermented bamboo powder could be one potential fiber-rich ingredient for growing–finishing pigs.

**Abstract:**

This experiment aimed to investigate the effects of fermented bamboo powder (FBP) on the growth performance, serum biochemical parameters, immunoglobulins and inflammatory cytokines, and fecal microbial composition of growing–finishing pigs. A total of 108 barrows (initial body weight, 56.30 ± 0.55 kg) were randomly allocated to three dietary treatments in a 75 d trial, including a control (CON) diet and two FBP supplementation diets. The CON diet was formulated to three-phase diets according to the body weight of pigs, and the FBP diets were formulated used 5.00% (FBP1) or 10.00% (FBP2) FBP to replace the wheat bran in the CON diet, respectively. The results showed that there were no influences on growth performances between the CON diet and FBP addition diets, whereas the 5% FBP addition decreased the feed:gain of pigs compared to the pigs fed the FBP2 diet from d 0–75 (*p* < 0.05). Meanwhile, the FBP addition increased the high-density lipoprotein cholesterol (HDLC) and immunoglobulin A (IgA) content in serum (linear, *p* < 0.05), and pigs fed the FBP1 diet had greater HDLC and IgA contents in serum than those in the pigs fed the CON diet (*p* < 0.05). Microbial analysis showed that the FBP addition diets decreased the abundance of Spirochaetes, and the FBP2 diet increased the abundance of Firmicutes more than the CON diet (*p* < 0.05). In addition, the pigs fed the FBP2 diet increased the abundance of *uncultured_bacterium_f_Lachnospiraceae*, *Ruminococcaceae_UCG-005*, *Prevotellaceae_UCG-003*, *Lachnospiraceae_XPB1014_group*, and *Lactobacillus* more than the CON group (*p* < 0.05). In conclusion, the FBP supplementation to the diet had no negative effects on the growth performance and exerted beneficial effects on promoting serum biochemical and immune indices, as well as modulating the fecal microbiota of pigs. Therefore, these results showed that the fermented bamboo powder could be one potential fiber-rich ingredient for growing–finishing pigs, and that the recommended addition proportion in the growing–finishing pigs’ diet is 5%.

## 1. Introduction

To solve the shortage of feed resources and alleviate the current situation of human and animal competition for food, there is a great need to find more sustainable feed materials for livestock production [1,2]. Fiber-rich ingredients as cost-effective alternative feedstuffs in swine diets have been observed over the last decade; however, the presence of lignin hinders their efficient utilization [3,4]. Therefore, pretreatment is required to open the complex network of lignocellulose and primarily to make the cellulose and hemicellulose amenable to microbiota or enzyme conversion [5]. Mechanical comminution and microbial fermentation are the most used pretreatment methods, where microbial fermentation pretreatment is under mild conditions and requires a lower energy consumption compared to mechanical pretreatment [6,7]. Additionally, fermentation could improve the nutritional values of common feed ingredients (such as soybean) or fiber-rich ingredients (such as corn-ethanol co-products or soybean hull) for monogastric animals [8,9].

Bamboo is a fast-growing perennial plant widely distributed in China and Southeast Asia areas and produces large amounts of biomass materials, thus having great potential to be used as a valuable feedstock for a variety of areas such as biomaterials, biofuel, and food, as well as for livestock production [10,11]. Previous studies showed that fresh bamboo and bamboo powder could be used as ruminant feed [12,13,14]. In addition, bamboo by-products such as bamboo charcoal, bamboo vinegar, and micronized bamboo powder could also be used as alternatives to antibiotics in the diet of pigs or broilers [15,16,17]. However, little is known about the fermented bamboo powder (FBP) used in swine diets. Therefore, the objective of this study was to evaluate the effects of graded levels of FBP on the growth performance, nutrient digestibility, serum antioxidant status, and fecal microbiota of growing–finishing pigs.

## 2. Materials and Methods

The experimental procedures were approved by the Experimental Animal Welfare and Ethical Committee of the Institute of Animal Science and Veterinary, Tianjin Academy of Agriculture Sciences. The FBP was kindly provided by Liuyang Huanan Bamboo Industry Company (Changsha, China), and was produced by *Yarrowia lipolytica* and *Lactobacillus plantarum* under solid-state fermentation. The analyzed nutrient composition is presented in Table 1.

### 2.1. Animals, Diets, and Experimental Design

A total of 108 Duroc × Yorkshire × Landrace crossbred barrows (initial body weight of 56.34 ± 0.65 kg) were randomly divided into 3 dietary treatments; each treatment contains 6 replicate pens and 6 pigs per pen. Pigs were housed in fully slatted pens and had free access to feed and water. Dietary treatments included a control diet (CON) and 2 FBP diets. The CON diet was composed of corn, wheat bran, soybean meal, and corn distillers’ dried grains with soluble (Table S1), whereas the FBP diets were formulated by replacing wheat bran in the three-phase CON diet with 5.0% (FBP1) or 10.0% FBP (FBP2), respectively. The nutrients of the three-phase CON diets were formulated to meet or exceed the nutrient requirements of growing–finishing pigs recommended by the National Research Council [18]. All pigs were allowed ad libitum access to feed and water throughout the 75 days experiment period. The room temperature and the relative humidity were controlled at 25 ± 2.5 °C and 60 ± 10% during the experimental periods.

### 2.2. Sample Collection

Each pen’s pigs’ body weight was recorded on day 0 and days 76 of the experiment, and feed consumption per pen was recorded at the end of the experiment to calculate average daily gain (ADG), average daily feed intake (ADFI), and feed-to-gain ratio (F:G).

On the morning of day 75, the fresh fecal samples of pigs were collected per pen in each dietary group to analyze the bacterial community, and were obtained using 5 mL centrifuge tubes, placed in liquid nitrogen, and then stored at a temperature of −80 °C.

The blood samples were collected from one pig from each pen via jugular vein puncture, and 5 mL was collected into a vacutainer on the morning of day 75. After 2 h, the blood samples were centrifuged at 1600× *g* at 4 °C for 15 min to recover serum, which was stored at −20 °C until analysis.

### 2.3. Chemical Analyses

The FBP and experimental diets were ground to pass through a 0.5 mm screen before analyses. The crude protein (method 990.03), ether extract (method 920.39), crude fiber (method 978.10), and ash (method 942.05) content of the diets and fecal samples were determined according to AOAC [19]. The gross energy content in diets and feces samples was analyzed using an adiabatic oxygen bomb calorimeter (model 6400, Parr Instruments, Moline, IL). Neutral detergent fiber and acid detergent fiber in the diets were determined using filter bags and fiber analyzer equipment (Fiber Analyzer; ANKOM Technology, Macedon, NY, USA) following a modification of the procedures [20]. After the samples underwent dry ashing at 550 °C for 4 h and wet digestion with nitric acids, the calcium contents were analyzed using EDTA titration method (method 967.30) according to AOAC [19]. The acid molybdate and Fiske–Subbarow reducer wet digestion solutions were added to the digested samples to determine phosphorus concentration by spectrophotometric reading of absorption at 620 nm (method 985.01) according to AOAC [19].

The alanine transaminase (ALT), aspartate transaminase (AST), total protein, total bilirubin, glucose, cholesterol, triglyceride, high-density lipoprotein cholesterol (HDLC), and low-density lipoprotein cholesterol (LDLC) in serum were measured from each sample using an automatic biochemical analyzer (Roche Hitachi 911 Chemistry Analyzer, Tokyo, Japan) according to the instructions their corresponding kits (Nanjing Jiancheng Bioengineering Company, Jiangsu, China). The serum levels of immunoglobulin A (IgA), immunoglobulin G (IgG), and immunoglobulin M (IgM) were measured by assay kits (Beijing Kangjiahongyuan Biotechnology Institute, Beijing, China). The cytokine levels of interleukin (IL)-4, IL-10, tumor necrosis factor (TNF)-α, and interferon (IFN)-γ in the serum were determined by enzyme-linked immunosorbent assay (ELISA) kits (Bio-function Technology Co., Ltd., Beijing, China) according to the instructions.

### 2.4. DNA Extraction and 16S rDNA Gene Sequencing

Fecal microbial DNA was isolated with DNA Kit (Omega, Bio-Tek, Norcross, GA, USA) according to the protocol of the manufacturer. The V3–V4 regions of bacteria 16S rDNA were amplified using the gene-specific primers 338F (5′-ACTCCTACGGGAGGCAGCAG-3′) and 806R (5′-GGACTACHVGGGTWTCTAAT-3′). The library was sequenced on the Illumina MiSeq platform (Illumina Inc., San Diego, CA, USA) according to the standard protocols by Biomarker Technologies Co., Ltd. (Beijing, China). Operational taxonomic units (OTUs) were clustered with a 97% similarity cutoff while using UPARSE. Then, chimeric sequences were identified and removed using UCHIME. The RDP database (http://rdp.cme.msu.edu/ (accessed on 21 April 2021)) was also referenced to take the taxonomy-based analysis for OTUs using the RDP classifier at a 90% confidence level. Taxonomy was assigned using the Silva (Release132 http://www.arb-silva.de (accessed on 1 April 2021)). The representative sequences of OTUs and their relative abundance were used to calculate the rarefaction analysis.

### 2.5. Statistical Analysis

The growth performance, digestibility of GE and nutrients of diets, and serum biochemical parameters were analyzed using the PROC GLM of SAS (Version 9.4, SAS Institute, Cary, NC, USA). Multiple comparisons were carried out with Duncan’s multiple comparison method at a 5% significant level. Orthogonal polynomial contrast was conducted to determine the linear and quadratic effects of the dietary FBP inclusion levels. A statistically significant difference was considered at *p* < 0.05.

Alpha-diversity, Chao1, ACE, Shannon, and Simpson’s evenness indexes were calculated at 97% identity. Beta-diversity was investigated with QIIME using principal component analysis (PCA) and principal coordinated analysis (PCoA) based on the weighted Unifrac distance matrix, and permutational multivariate analysis of variance (PERMANOVA) was calculated to determine significant differences in the microbial community. Statistical differences in phyla and genera among the groups were calculated by the Kruskal–Wallis rank-sum test with a corrected *p*-value < 0.05 (using Storey-FDR) representing statistical significance. The linear discriminant analysis (LDA) effect size (LEfSe) analysis using the non-parametric factorial Kruskal–Wallis sum-rank test was performed to find out the biomarker species in each group and characterize the difference (LDA score ≥ 4). The Spearman correlation analysis was applied to analyze the associations of the differential microbial species with the measured parameters.

## 3. Results

### 3.1. Growth Performance

There was no difference in the initial BW, ADG, and ADFI among the dietary treatments (Table 2). There was a linear effect of FBP on the final BW (*p* < 0.05), although ANOVA showed a tendency. The F:G was affected by the FBP levels: quadratically *(p* < 0.05), a 5% FBP addition decreased the feed:gain of the pigs compared to the pigs fed the FBP2 diet from d 0–75 (*p* < 0.05), whereas there was no difference in the F:G of pigs between the CON group and the FBP addition groups.

### 3.2. Serum Biochemical Parameters, Immunoglobulins, and Inflammatory Cytokines

The ALT, AST, total protein, total bilirubin, glucose, cholesterol, triglyceride, and LDLC contents in serum were not affected by the dietary treatments (Table S2). The HDLC content in serum was linearly increased with the FBP addition levels in the dietary treatments, and the FBP1-fed pigs had a greater HDLC content in serum than those in the pigs of the CON group (*p* < 0.05).

The results of serum immunoglobulins and inflammatory cytokines are shown in Table 3. No difference was observed in the contents of IgG, IgM, IL-4, IL-10, TNF-α, and IFN-γ in serum among the four dietary treatments. However, the IgA content in serum increased linearly with an increased inclusion level of FBP, and the 5% FBP supplementation had a greater IgA content in the serum of pigs than in the CON group (*p* < 0.05).

### 3.3. Fecal Bacterial Community

**Figure 1:**
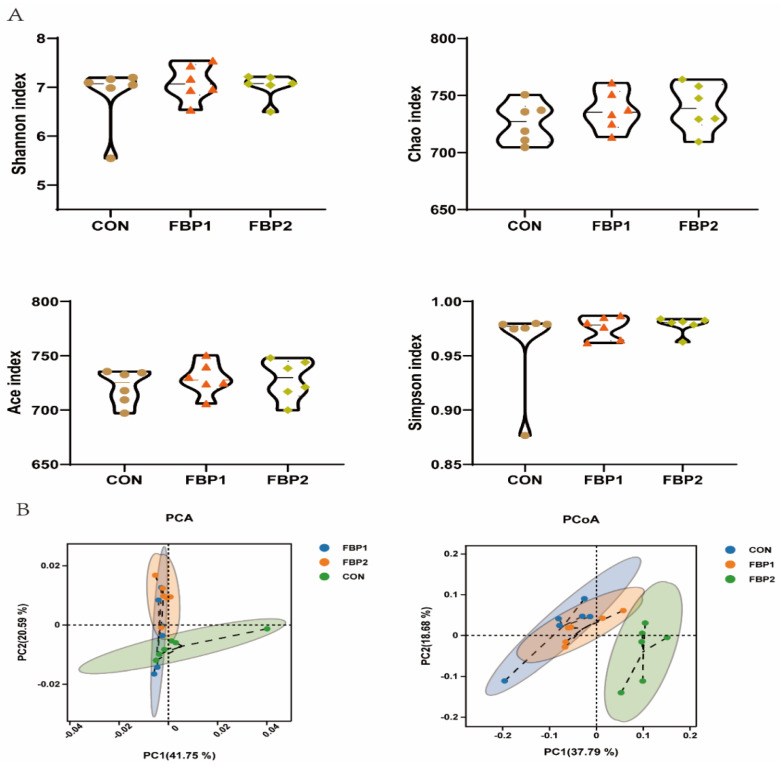
Diversity analyses of microbial communities among dietary groups. (**A**) Alpha diversity analysis. (**B**) Beta diversity with principal component analysis (PCA) and principal coordinates analysis (PCoA) based on unweighted Unifrac distance. CON, control diet. FBP1, the 5% fermented bamboo powder supplementation diet. FBP2, the 10% fermented bamboo powder supplementation diet.

The rate of emergence of new OTUs in this experiment tended to level off under continuous sampling, demonstrating that the number of species in this experiment did not increase significantly with the number of sequencings (Figure S1). Four α diversity indices of fecal bacteria were calculated, the Chao index and Ace index were used to estimate species richness, and Shannon and Simpson diversity values were used to estimate the microbial diversity in the samples. There was no difference in the species richness and microbial diversity in the samples among the three dietary treatments (Figure 1A). Regarding the fecal bacterial composition in terms of β-diversity clustering by PCA and PCoA based on the weighted Unifrac distance matrix, the results revealed a significant separation of fecal microbiome between the CON and FBP2 groups (Figure 1B).

**Figure 2:**
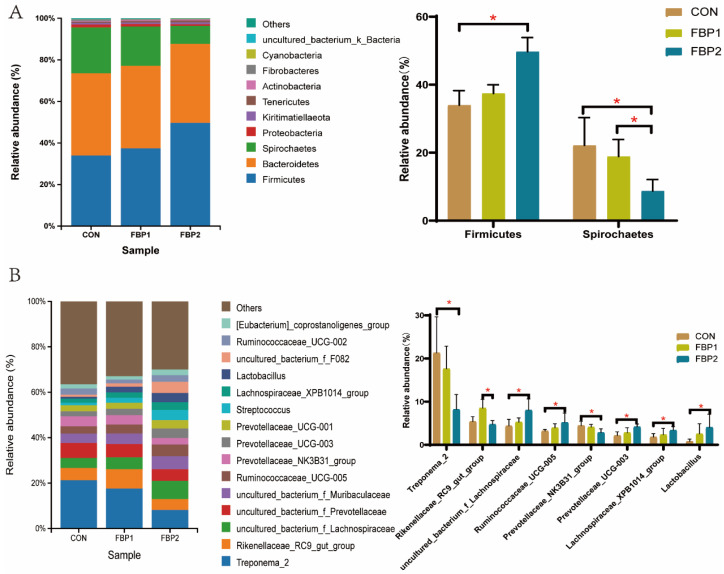
Composition of the bacteria at the level of phylum (top 10, (**A**)) and genus (top 15, (**B**)). The left side of the figure shows the composition of the bacteria at each level, and the right side shows the differential bacteria at each level. CON, control diet. FBP1, the 5% fermented bamboo powder supplementation diet. FBP2, the 10% fermented bamboo powder supplementation diet. * indicates statistically significant difference (*p* < 0.05).

The composition of the top 10 phyla and top 15 genera in the fecal contents of pigs is shown in Figure 2. At the phylum level, the *Firmicutes*, *Bacteroidetes*, and *Spirochaetes* were the dominant bacteria, and accounted for more than 94% of the whole phyla (Figure 2A). The pigs in the FBP2 group had a higher relative abundance of *Firmicutes* compared with the CON group (*p* < 0.05, Figure 2A); however, they had a lower abundance of *Spirochaetes* compared with the CON and FBP1 group (*p* < 0.05, Figure 2A). At the genus level, the dominant microbiota consisted of *Treponema_2*, *Rikenellaceae_RC9_gut_group*, *uncultured_bacterium_f_Lachnospiraceae*, *uncultured_bacterium_f_Prevotellaceae*, *uncultured_bacterium_f_Muribaculaceae*, *Ruminococcaceae_UCG-005, Prevotellaceae_NK3B31_group*, *Prevotellaceae_UCG-003*, *Prevotellaceae_UCG-001*, *Streptococcus*, *Lachnospiraceae_XPB1014_group*, and *Lactobacillus*, which contained approximately 60% of the total sequences (Figure 2B). The 5% FBP supplementation increased the abundance of *Rikenellaceae_RC9_gut_group* compared with the FBP2 group (*p* < 0.05, Figure 2B). Compared to the CON group, the 10% FBP addition group had an increased abundance of *uncultured_bacterium_f_Lachnospiraceae*, *Ruminococcaceae_UCG-005*, *Prevotellaceae_UCG-003*, *Lachnospiraceae_XPB1014_group*, and *Lactobacillus*, whereas it had a decreased abundance of *Treponema_2* and *Prevotellaceae_NK3B31_group* (*p* < 0.05, Figure 2B).

**Figure 3:**
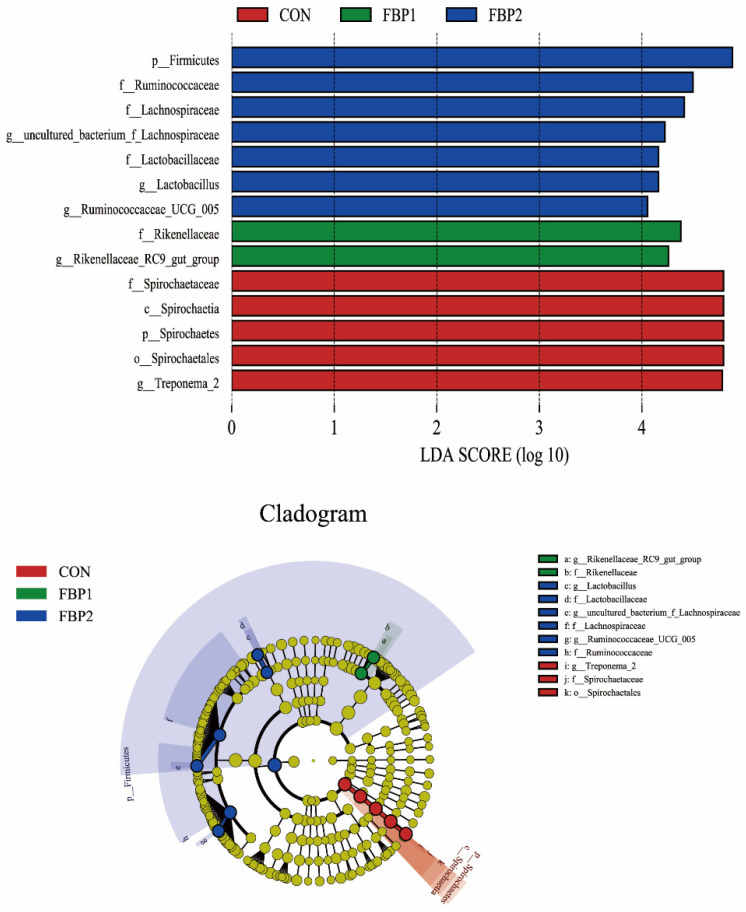
Linear discriminant analysis effect size (LEfSe) analysis (*p* < 0.05, LDA > 4.0) of the fecal microbes. The prefixes “p”, “c”, “o”, “f”, and “g” represent the annotated level of phylum, class, order, family, and genus. CON, control diet. FBP1, the 5% fermented bamboo powder supplementation diet. FBP2, the 10% fermented bamboo powder supplementation diet.

The LEfSe analysis identified 14 biomarkers with LDA scores > 4. The results showed that seven bacterial taxa were found to be enriched in the FBP2 group, including *Firmicutes* (phylum), *Ruminococcaceae* (family), *Lachnospiraceae* (family), *Lactobacillaceae* (family), *Lactobacillus* (genus), *uncultured_bacterium_f_Lachnospiraceae* (genus), and *Ruminococcaceae_UCG-005* (genus). Two bacterial taxa were found to be enriched in both FBP1 groups: were *Rikenellaceae* (family) and *Rikenellaceae_RC9_gut_group* (genus). In addition, *Spirochaetes* (phylum), *Spirochaetia* (class), *Spirochaetales* (order), *Spirochaetaceae* (family), and *Treponema_2* (genus) were enriched in the CON group (Figure 3).

**Figure 4:**
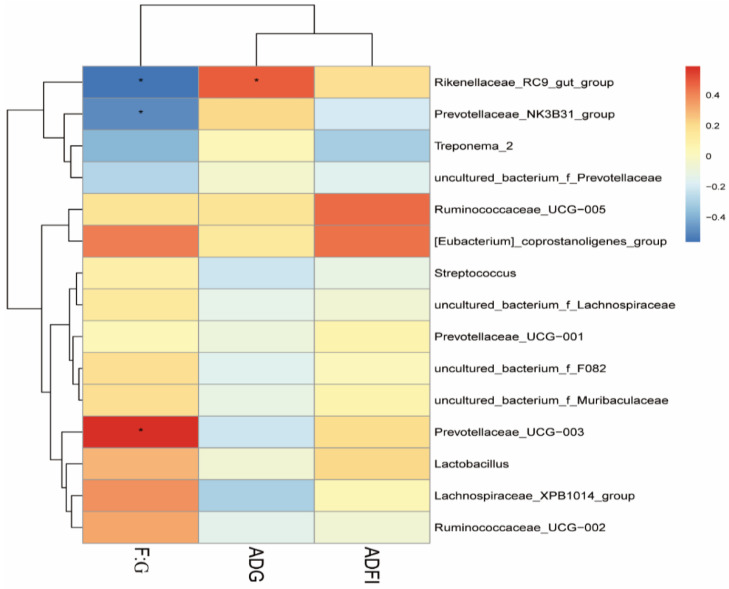
The Spearman correlation analysis of fecal microbial species at genus level with growth performance of pigs. Spearman correlation coefficients are represented by color ranging from red, positive correlation (0.6), to blue, negative correlation (−0.6). * indicates statistically significant difference (*p* < 0.05). ADFI, average daily feed intake. ADG, average daily gain. F:G, feed-to-gain ratio.

The results of Spearman’s correlation analysis between growth performance from day 1 to 75 and the top 15 genera in the pigs are presented in Figure 4. The average abundance of the genera *Rikenellaceae_RC9_gut_group* and *Prevotellaceae_NK3B31_group* were negatively correlated with F:G, whereas the genera of *Prevotellaceae_UCG-003* were positively correlated with F:G (*p* < 0.05). Additionally, the average abundance of *Rikenellaceae_RC9_gut_group* was also positively correlated with ADG (*p* < 0.05).

## 4. Discussion

Fiber-rich ingredients as alternative feedstuffs in swine diets have been observed over the last decade, and were thought to be an effective way to reduce feed costs and improve the gut health of pigs [4,21]. Bamboo is a fast-growing perennial plant, and bamboo powder contains abundant dietary fiber; therefore, it was considered an important alternative feed feedstock in ruminants’ production [11,12,13]. However, there is still a need for more research to study the application of FBP in swine production. There were no negative influences in the growth performance, such as ADG and ADFI, for growing–fattening pigs fed the FBP addition diets during the experimental period in the current study. In addition, the 5.0% FBP addition diet decreased the F:G compared to the pigs fed the 10% FBP addition diet. This was consistent with the previous study reported by Dai, et al. [22], where the weaned piglets fed diets with 1% micronized bamboo powder addition increased their ADG but decreased their F:G compared with the CON diet. Similarly, another study revealed that 1% micronized bamboo powder could also improve the growth performance of broilers in an antibiotic-free basal diet [16]. These results indicated that bamboo powder could be considered as a source of feed ingredients in monogastric livestock (pigs and chickens) production, and wheat bran could be replaced by FBP in the diets of growing–fattening pigs.

The serum biochemical parameters, immunoglobulins, and inflammatory cytokines were usually used in the nutritional assessment to determine the quality of test feedstuffs or additives to reflect the physiological, metabolic, and humoral immunological status of animals [23,24,25]. In the current study, the addition of FBP had no effects on most serum biochemical parameters and inflammatory cytokines. However, the 5% FBP addition increased the contents of HDLC in the serum more than in the CON group. A previous study showed that serum HDLC levels are inversely related to cardiovascular disease risk, obesity, and metabolic syndrome [26]. Agreed with the previous study that the addition of bamboo products increased the concentrations of IgA in serum [15], while the IgA could play a role against bacterial or viral infections, both in serum and mucosal secretions [27]. Therefore, the increased HDLC and IgA resulting from FBP addition may improve the health condition of pigs, as well as protect pigs from diseases. The increased contents of HDLC and IgA may be attributed to the richness of flavonoids, organic acids, phenolic compounds, and polysaccharides in bamboo products, which can regulate the lipid metabolism and immune function of animals [28,29].

The microbial community in the animal gut plays a critical role in intestinal morphology, nutrition digestion, and host immune system modulation [30]. Previous studies demonstrated that the addition of fermented feed has a positive effect on modulating the intestinal microbiota, which could promote the presence of beneficial bacteria and reduce potentially harmful bacteria populations in the gut of pigs [31]. Additionally, bamboo powder is a kind of fiber-rich ingredient, which could also influence the composition and functionality of the gut microbiota [32]. In the current study, the FBP addition had no effects on the overall microbial diversity in the feces but did alter the composition of intestinal bacterial microbiota in pigs. The LefSe results showed that FBP addition induced differentially enriched bacterial species at different taxonomic levels. At the genus level, the 5% FBP addition group enriched *Rikenellaceae_RC9_gut_group*, and the potential biomarkers in the 10% FBP addition group were *uncultured_bacterium_f_Lachnospiraceae*, *Lactobacillus*, and *Ruminococcaceae_UCG-005*. It was reported that these four bacteria could degrade carbohydrates (such as indigestible oligosaccharide, cellulose, hemicellulose, or resistant starch) and produce acetate, butyrate, or lactic acid. It can be inferred that they can improve the host’s nutrient digestion and absorption through carbohydrate metabolism in FBP-fed pigs [33,34,35]. However, the LefSe results suggested that the potential pathogens (*Treponema_2*) may be the biomarker of the control group. These results implied that the FBP supplementation may exert beneficial effects on modulating the intestinal microbiota of pigs. *Rikenellaceae_RC9_gut_group* is one genus of bacteria *Bacteroidales*, and could degrade cellulose and hemicellulose that enhanced the fiber digestion [34,36]. The Spearman correlation analysis in our study showed that *Rikenellaceae_RC9_gut_group* was negatively correlated with FC but positively correlated with ADG, suggesting that the low F:G in the 5% FBP addition group may be attributed to the increased abundance of *Rikenellaceae_RC9_gut_group*. Therefore, further studies are required to explore the evolution of the intestinal microbiota in FBP-supplemented pigs and uncover the relationship between the *Rikenellaceae_RC9_gut_group* and the growth performance of growing–finishing pigs.

## 5. Conclusions

In conclusion, the FBP supplementation to the diet had no negative effects on the growth performance and exerted beneficial effects on promoting serum biochemical and immune indices, as well as modulating the fecal microbiota of pigs. Therefore, these results indicated that fermented bamboo powder could be one potential fiber-rich ingredient for growing–finishing pigs, and that the recommended addition proportion in the growing–finishing swine diet is 5%.

## Figures and Tables

**Table 1:** Analyzed composition of the fermented bamboo powder (%, as-fed basis).

Item	Fermented Bamboo Powder
Dry matter	89.80
Crude protein	2.28
Ether extract	0.18
Neutral detergent fiber	60.80
Acid detergent fiber	47.36
Calcium	0.12
Total phosphorus	0.02
Gross energy, MJ/kg	16.85

**Table 2:** Effects of fermented bamboo powder supplementation on growth performance of growing–finishing pigs.

Item ^1^	CON	FBP1	FBP2	SEM	*p*-Value		
					ANOVA	Linear	Quadratic
Initial body weight, kg	56.28	56.47	56.28	0.15	0.864	0.649	0.782
Final body weight, kg	131.80	132.81	131.66	0.21	0.052	0.047	0.131
Average daily gain, kg	1.03	1.05	1.03	0.00	0.265	0.197	0.315
Average daily feed intake, kg	2.91	2.93	2.94	0.01	0.438	0.316	0.426
Feed:gain	2.81 ^a,b^	2.80 ^b^	2.84 ^a^	0.01	0.016	0.479	0.005

^1^ CON, control diet; FBP1, the 5% fermented bamboo powder supplementation diet; FBP2, the 10% fermented bamboo powder supplementation diet; SEM, standard error of mean. ^a,b^ Values in the same row with different superscript letters were significantly different (*p* < 0.05).

**Table 3:** Effects of fermented bamboo powder supplementation on serum immunoglobulins and inflammatory cytokines of growing–finishing pigs.

Item ^1^	CON	FBP1	FBP2	SEM	*p*-Value		
					ANOVA	Linear	Quadratic
IgG, g/L	8.71	8.78	8.62	0.15	0.925	0.875	0.722
IgA, g/L	0.97 ^b^	1.27 ^a^	1.04 ^a,b^	0.05	0.046	0.019	0.401
IgM, g/L	0.78	0.75	0.76	0.01	0.586	0.369	0.625
IL-4, pg/mL	23.66	30.19	21.41	2.88	0.482	0.390	0.401
IL-10, pg/mL	17.80	28.71	8.85	5.19	0.334	0.412	0.218
TNF-α, pg/mL	36.38	35.45	19.19	6.28	0.502	0.955	0.249
IFN-γ, pg/mL	47.73	42.89	34.09	2.91	0.172	0.496	0.082

^1^ CON, control diet; FBP1, the 5% fermented bamboo powder supplementation diet; FBP2, the 10% fermented bamboo powder supplementation diet; SEM, standard error of mean; IgG, immunoglobulin G; IgA, immunoglobulin A; IgM, immunoglobulin M; IL-4, interleukin-4; IL-10, interleukin-10; TNF-α, tumor necrosis factor-α; IFN-γ, interferon-γ. ^a,b^ Values in the same row with different superscript letters were significantly different (*p* < 0.05).

## Data Availability

The data that support the findings of this study are available from the corresponding author upon reasonable request.

## Supplementary Materials

The following supporting information can be downloaded at: https://www.mdpi.com/article/10.3390/ani12223127/s1. Table S1. Ingredients composition and nutrient values of control diets; Table S2. Effects of fermented bamboo powder supplementation on serum biochemical parameters of growing-finishing pigs, Figure S1. Rarefaction curves. The horizontal coordinate is the number of randomly selected sequencing strips, and the vertical coordinate is the number of features obtained based on the number of sequencing strips. Each curve represents one sample, marked with different colors. CON, control diet; FBP1, the 5% fermented bamboo powder supplementation diet; FBP2, the 10% fermented bamboo powder supplementation diet.
