# Supplementary material for: Effects of Fermented Bamboo Powder Supplementation on Serum Biochemical Parameters, Immune Indices, and Fecal Microbial Composition in Growing–Finishing Pigs
Source: Animals (Basel). 2022 Nov 13;12(22):3127. doi: 10.3390/ani12223127 (PMC9686535; doi:10.3390/ani12223127)
Supplement: Supplementary file 1 [file animals-12-03127-s001.zip › animals-1958884-supplementary.pdf]

Table S1. Ingredients composition and nutrient values of control diets (% as fed basis).

Item	Phase 1: 50 – 75 kg	Phase 2: 75 – 100 kg	Phase 3: >100 kg
Corn	60.51	63.77	67.06
Soybean meal	13.00	9.00	5.00
Corn distillers dried grains with soluble	5.00	5.00	5.00
Wheat bran	15.00	16.00	17.00
Soybean oil	3.00	3.00	3.00
Dicalcium phosphate	0.50	0.35	0.10
Limestone	1.00	1.00	1.00
Calcium formate	0.10	0.10	0.10
Salt	0.30	0.30	0.30
Choline chloride	0.10	0.10	0.10
L-Lysine-HCL	0.33	0.30	0.28
DL-Methionine	0.03	0.02	0.00
L-Threonine	0.10	0.08	0.06
Phytase	0.03	0.03	0.03
Premix[1]	1.00	1.00	1.00
Total	100.00	100.00	100.00
Nutrient values[2]			
Metabolizable energy, MJ/Kg	14.21	13.64	13.05
Crude protein	14.27	12.95	11.83
Neutral detergent fiber	14.13	16.35	18.69
Acid detergent fiber	5.32	7.11	8.94
Calcium	0.58	0.53	0.46
Total phosphorus	0.52	0.48	0.43
Lys	0.97	0.84	0.72
Thr	0.64	0.56	0.49
Met+Cys	0.57	0.50	0.43

[1] The premix provides the following per kg of diet: 6500 IU of vitamin A, 2400 IU of vitamin D_3, 20 mg of vitamin E, 2.4 mg of vitamin K_3, 2.4 mg of vitamin B_1, 6.6 mg of vitamin B_2, 3 mg of vitamin B_6, 0.025 mg of vitamin B_{12}, 25 mg of nicotinic acid, 13 mg of pantothenic acid, 0.2 mg of biotin, 15 mg of Cu (as $CuSO_4$), 150 mg of Fe (as Fe_2SO_4), 80 mg of Zn (as ZnO), 50 mg of Mn (as $MnSO_4$), 0.6 mg of I (as KI), and 0.3 mg of Se (as Na_2SeO_3).
[2] Nutrient values were analyzed except for metabolizable energy.

Table S2. Effects of fermented bamboo powder supplementation on serum biochemical parameters of growing-finishing pigs.

Item[1]	CON	FBP1	FBP2	SEM	P-value		
					ANOVA	Linear	Quadratic
ALT, U/L	44.50	45.67	46.67	3.76	0.977	0.909	0.859
AST, U/L	40.50	42.83	40.67	3.14	0.953	0.785	0.893
Total protein, g/L	59.95	58.25	60.73	3.04	0.953	0.837	0.820
Total bilirubin, mmol/L	0.40	0.28	0.43	0.08	0.778	0.604	0.638
Glucose, mmol/L	3.42	3.58	3.40	0.10	0.741	0.534	0.655
Cholesterol, mmol/L	2.13	1.61	1.88	0.11	0.192	0.074	0.950
Triglyceride, mmol/L	0.45	0.39	0.41	0.03	0.647	0.362	0.920
HDLC, mmol/L	0.71b	0.92a	0.81a,b	0.03	0.004	0.001	0.830
LDLC, mmol/L	1.07	1.00	1.03	0.07	0.931	0.712	0.973

[1] CON, control diet; FBP1, the 5% fermented bamboo powder supplementation diet; FBP2, the 10% fermented bamboo powder supplementation diet; SEM, standard error of mean; ALT, alanine transaminase; AST, aspartate transaminase; HDLC, high-density lipoprotein cholesterol; LDLC, low-density lipoprotein cholesterol.

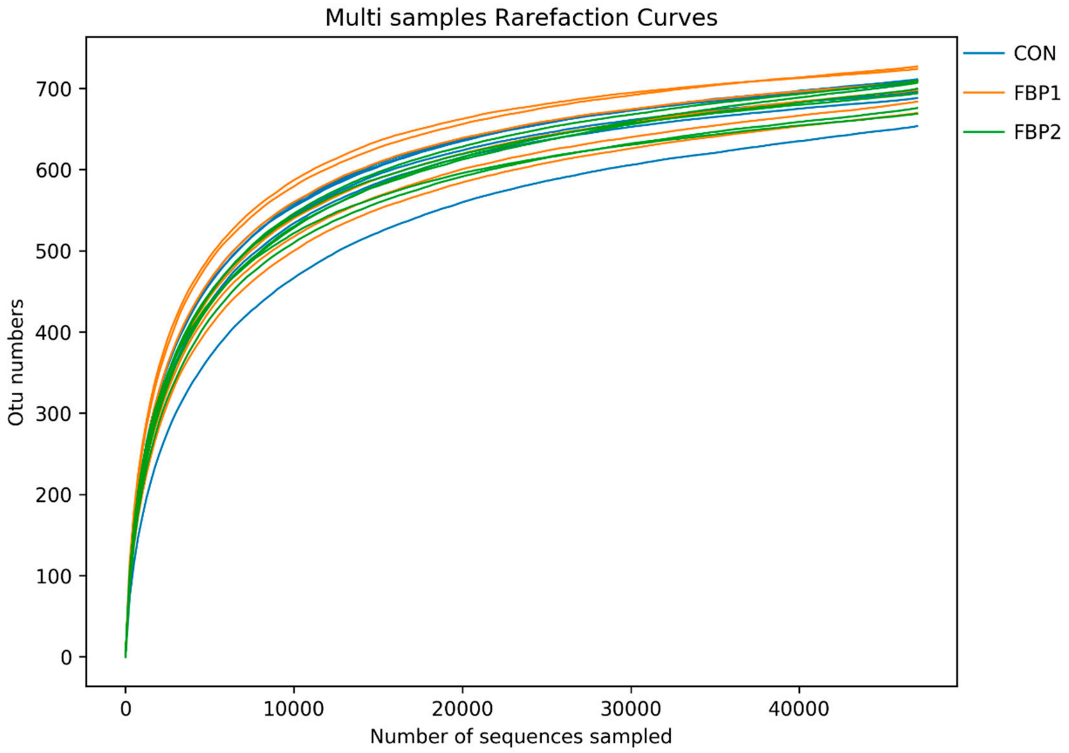

Figure S1. Rarefaction curves. The horizontal coordinate is the number of randomly selected sequencing strips, and the vertical coordinate is the number of features obtained based on the number of sequencing strips. Each curve represents one sample, marked with different colors. CON, control diet; FBP1, the 5% fermented bamboo powder supplementation diet; FBP2, the 10% fermented bamboo powder supplementation diet.
